# Correlation Between West Nile Virus and Pregnancy: A Systematic Review

**DOI:** 10.3390/pathogens13121129

**Published:** 2024-12-20

**Authors:** Maria Marnezi, Aristotelis Tsiakalos, Karolina Akinosoglou

**Affiliations:** 1 Hellenic Open University, 26335 Patra, Greece; std531606@ac.eap.gr; 2 Lito General, Maternity & Gynecology Clinic, 11524 Athens, Greece; 3 Department of Medicine, School of Health Sciences, University of Patras, 26504 Rio, Greece; akin@upatras.gr

**Keywords:** West Nile virus, pregnancy, pregnant, neonates, breast milk

## Abstract

Background: West Nile Virus [WNV] is a mosquito-borne flavivirus. It has spread globally, causing asymptomatic to severe neurological diseases in humans, with an increased risk in older adults and those with underlying conditions. This review examines WNV’s impact on pregnancy, focusing on maternal and neonatal symptoms and risks. Methods: This systematic review included primary studies from “PUBMED” and “SCOPUS” databases, as well as Google and Google Scholar, conducted in July 2024 using the appropriate keywords. This review adhered to PRISMA guidelines and utilized the Newcastle–Ottawa scale for bias assessment. Results: Seven primary studies were included in the systematic review. Fever was the predominating symptom, including neurological manifestations, respiratory symptoms, myalgia, weakness, nausea, vomiting, and rashes. Delivery, in most cases, progressed without any complications, while no infection was noted. Most of the neonates had a normal Apgar score, and their developmental functions did not seem to be affected. Even though, antibodies against WNV were detected in breast milk, no association with transmission to the neonate was observed. Conclusions: WNV infection is mostly associated with favorable outcomes during pregnancy. However, larger cohorts are needed to confirm our conclusions. Prompt diagnosis and public health surveillance are pivotal to eliminate disease transmission.

## 1. Introduction

The West Nile Virus (WNV) is a mosquito-borne flavivirus that has emerged as an important global public health issue. First identified in Uganda in 1937, the virus has spread since then to various parts of the world, including Africa, Europe, Asia, and the Americas [1]. According to the WHO, it is considered the leading cause of mosquito-borne disease in the continental USA [2,3]. The main vectors for WNV are *Culex* species of mosquitoes, which transmit the virus to birds, which are the primary reservoir hosts, and on many occasions, to humans and other mammals. Among the birds, *Turdus migratorius* and *Passer domesticus* are the most important species that reside in European and North American cities [4,5]. Clinical manifestations of WNV infection in humans can vary from asymptomatic to severe neurological disease. Approximately 80% of infected individuals remain asymptomatic, while 20% present symptoms such as fever, headache, body aches, rash, vomiting, and fatigue [6]. However, less than 1% of the infected individuals develop severe neuroinvasive disease that presents with fever in conjunction with encephalitis, meningitis, flaccid paralysis, or mixed symptoms [7]. The risk of severe disease increases with age, genetic factors, and underlying medical conditions, such as hematological malignancies in particular and organ transplantation [8]. Diagnosis of WNV infection is primarily based on the detection of virus-specific antibodies in the serum or cerebrospinal fluid [9]. IgM antibodies, which appear early in the course of infection, are indicative of recent exposure, whereas IgG antibodies suggest past infection [10]. Molecular techniques such as reverse transcription-polymerase chain reaction [RT-PCR] can also be used to detect viral RNA in clinical samples [11]. Until recently, there has been no specific treatment for WNV infection. Management is largely supportive, focusing on alleviating symptoms and preventing complications [3]. In severe WNV infection, hospitalization and intensive care may be required. Efforts to develop vaccines are ongoing, with several candidates showing promising results in preclinical and clinical trials [12].

The recent outbreak of emerging viruses, including severe acute respiratory syndrome coronavirus 2 (SARS-CoV-2), has highlighted concerns about the effects of viral infections on the health of both mothers and their unborn children during pregnancy [13]. During pregnancy, various physiological changes, particularly in the immune and endocrine systems, take place, making both the mother and fetus more prone to certain viral and bacterial infections, increasing the likelihood of severe complications from these diseases [14]. Adverse outcomes of viral infections in pregnancy include maternal illness, miscarriage, stillbirth, intrauterine growth restriction (IUGR), premature birth, neonatal death, and congenital abnormalities [14,15]. Due to the potential safety and toxicity concerns surrounding the use of antiviral treatments and vaccines during pregnancy, pregnant women are considered even more susceptible to the severe effects of viral infections.

Viral infection during pregnancy can have serious consequences for both newborns and fetuses [16]. Intrauterine fetal infection with various flaviviruses is frequently linked to fetal death, miscarriage, preterm delivery of stillborns, or the death of newborns shortly after birth. However, most infants appear to lead a seemingly normal life [17]. Several flaviviruses, including Zika virus (ZIKV), WNV, and Powassan virus (POWV), have been shown to infect the placenta and cause fetal death in both mouse models and human placental explants [18]. These neurotropic viruses are capable of crossing the placental barrier, infecting the fetal central nervous system, and leading to brain damage [18]. Maternal inflammatory responses triggered by these infections can impair fetal neurodevelopment, raising the risk of conditions such as microcephaly, autism, and cerebral palsy [19]. Clinical findings confirm transplacental transmission of flaviviruses like ZIKV and dengue virus (DENV) [20]. Additionally, viruses such as parvovirus B19 and cytomegalovirus have been linked to cases of fetal loss, underscoring the importance of regular screening and preventive measures [21].

Data remain scarce as to WNV infection in pregnancy [22], although it has been well established that human placental extravillous trophoblasts (EVTs) are permissive to WNV infection, which may disseminate the virus to the fetus [23]. Nonetheless, in contrast to other viruses, mother–fetal transmission and severe impact on newborns seem to be extremely rare in the case of WNV infection [17]. That said, we aimed to explore the impact of WNV in pregnancy, including risks, manifestations, and outcomes for both pregnant women and newborns.

## 2. Materials and Methods

**Figure 1:**
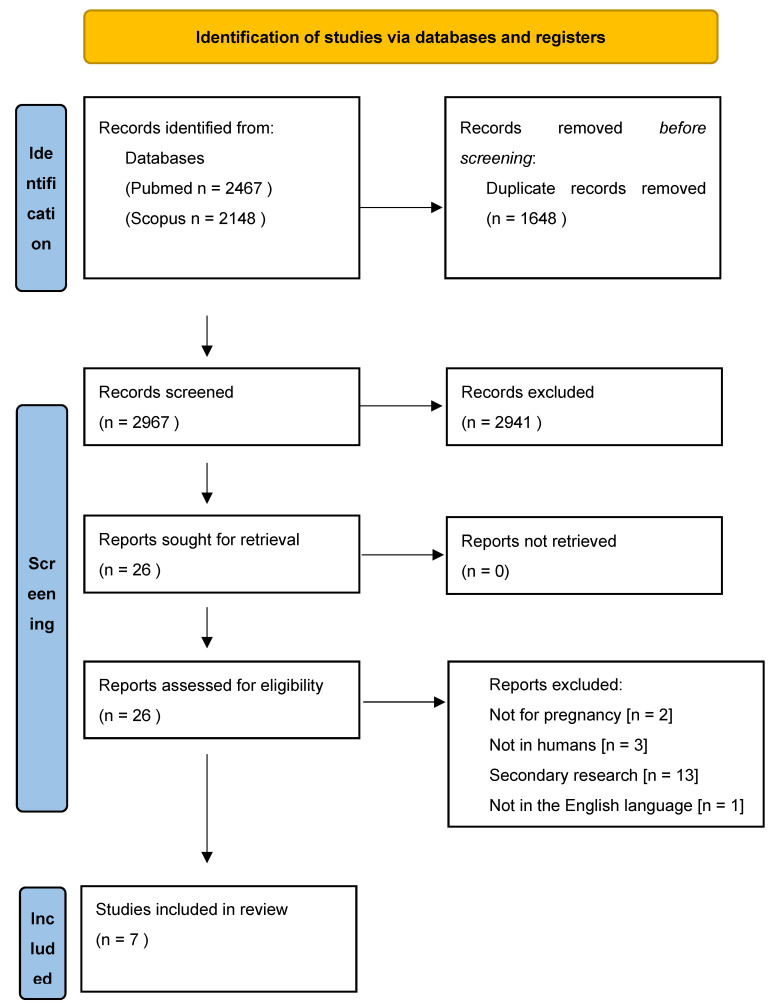
PRISMA flow diagram.

A systematic search was performed for primary studies that were published in the “PUBMED” and “SCOPUS” databases, as well as a manual search in Google and Google Scholar. The search was conducted in July 2024 with the relevant keywords, as shown below. More specifically, the keywords used were “West Nile Virus”, “WNV”, “flavivirus”, “arbovirus”, “pregnancy” and “embryo”, and “newborn” and “neonate”. Only English language papers pertaining to human subjects were included. No time limit was placed on the type of published studies. Primary studies resulting from the search were judged based on the PRISMA [Preferred Reporting Items for Systematic Reviews and Meta-Analyses] Statement tool, which is a flow chart for the precise identification of studies assessed for inclusion in the review without entering into a qualitative assessment of the primary studies (Figure 1) [24].

The studies used in the review were then assessed using the Newcastle–Ottawa scale for bias (Table 1). In statistical studies, this scale is used to assess the quality of non-randomized studies used in systematic reviews and meta-analyses.

Based on this tool, studies are judged on eight axes, categorized into three groups: selection of groups, comparability of groups, and ascertainment of either the exposure or the outcome of interest for case or cohort studies, respectively. The stars awarded for each type of review serve as a quick visual assessment. Stars are awarded so that the highest-quality studies receive up to nine stars. Finally, the primary studies were separated according to the axis on which they were categorized in order to make the appropriate use for processing the results [25].

## 3. Results

Upon reviewing the articles, seven primary studies were included in the systematic review [26,27,28,29,30,31,32] (Table 2). In brief, all studies were conducted in the U.S.A. Regarding study types, two were case studies, four were cross-sectional studies, and one was a prospective study [26,27,28,29,30,31,32]. One study examined the frequency of WNV infection during pregnancy and intrauterine WNV infection [29], four studies examined the effect of WNV during pregnancy [26,30,31,32], one study examined the effects during lactation [28], and finally, one study examined the effects on newborn development up to 3 years of age [27]. The summary of the included studies is presented in Table 2.

In terms of pregnant mothers, Pridjian et al. reported that, there were no statistically significant differences between the pregnancies with WNV and not, except that mothers with infections were more likely to have a fever and used a greater amount of medication [26]. However, in a case study by Stewart et al., 33.3% presented with neurological symptoms [32], similar to a previous case study of an African-American woman infected with the WNV during the second trimester of pregnancy presenting with encephalitis [31].

In terms of transmission, in the earliest cross-sectional study, conducted by Paisley et al. in 2005, examining 563 women infected with the WNV and their newborns [29], no statistically significant difference in neonates according to maternal disease was noted, while intrauterine WNV infections seemed to be infrequent. The 2003 study by Hinckley et al. investigated the breast milk of six newborns breastfed by mothers affected by the WNV [28]. Of those, five remained healthy, and one developed a rash [28]. Two fell ill during breastfeeding, and two had congenital antibodies [28].

As to birth outcomes, O Leary et al. monitored the outcomes of seventy-seven pregnant women with WNV [30]. A total of 71 delivered 72 live infants, while 4 had miscarriages, and 2 had elective abortions. Most infants were born at term, while abnormal growth was noted in 8 infants [30]. Preterm birth, low birth weight, and any major defects were noted in 5.6, 4.8, and 10.6% of newborns. Pridjian et al., nonetheless [26], observed similar birth weight characteristics regarding weight, length, head circumference, and rate of congenital malformation in babies born to WNV-infected versus uninfected mothers [26]. Of note, their prospective study involved 28 pregnant women infected with the virus and 25, matched on maternal age and enrollment month, uninfected women serving as controls and 58 neonates [26].

At their follow-up physical exams and Bayley II assessment scores, children born to mothers with WNV showed performance at or above age level across domains [26], in line with a cross-sectional study of 11 neonates performed in 2014, showing no developmental delays in young children [27].

## 4. Discussion

In the present systematic review, we attempted to study the effect of WNV on pregnancy. The aim was to record the symptoms of WNV infection for both mother and fetus or newborn, as well as the possible risks that have been recorded for pregnant women and newborns. We identified seven studies with variable results, in line with diverse study designs.

A constellation of clinical manifestations has been described during WNV disease in pregnant women, with disease onset occurring mostly during the second and third trimesters [25], but not always [30]. Fever emerged as a predominant symptom, occurring in five out of seven studies [26,28,29,30,31,32]. Interestingly, frequency varied from 18–100% among the populations included in these studies. Other notable symptoms included neurological manifestations, reported in three of the five studies, followed by respiratory symptoms [32]. Additional symptoms comprised myalgia, headaches, weakness, difficulty walking, chills, nausea, vomiting, and rashes [26,27,28,29,30,31,32]. Neurological symptoms, suggesting neuroinvasive disease, were reported in Chapa et al.’s study, including cervical stiffness, diplopia, and decreased joint movement, with the patient’s condition worsening during hospitalization [31]. Pridjian et al. reported meningitis or encephalitis in 11 out of 28 participants [26], and 23% of participants in O’Leary et al.’s study exhibited neurological symptoms [30]. Notably, only one pregnant woman required further medical intervention [30]. Pregnant women’s symptoms and prognosis match those of the general population, as they are mentioned in the study by Kramer et al. [33]. A noteworthy difference is that there was a zero percent mortality following neuroinvasive disease compared to the average 10% of the general population, a finding that needs further investigation to be elucidated [34]. Although mothers who contracted the virus used more medication during pregnancy than uninfected mothers, no significant differences in pregnancy complications between the two groups were noted [26]. Conversely, in mice, pregnancy seems to increase mortality risk independent of the infecting dose or the week of pregnancy [35]. However, the immunological response, as reflected in antibody titers, does not seem to affect the outcome [35]. The susceptibility of inbred mice to WNV infection has been associated with point mutations in the 2′-5′-oligoadenylate synthetase gene [36]. Since interferon regulation can be altered during pregnancy, these mutations could partially explain the differences observed between mice and humans.

Almost all included studies verified the existence of the WNV disease, through virus-specific immunoglobulin M (IgM) and/or immunoglobulin G (IgG) antibodies [26,27,28,29,30,31,32]. IgM antibodies typically appear within 3 to 4 days of symptom onset, with almost all patients having detectable levels by day 7 to 8 [37]. IgG antibodies are generally detectable by around 3 weeks after infection [37], and both IgM and IgG can persist for over a year [37,38]. In pregnant women, 4% of cord blood samples tested positive for WNV-specific IgG, but none had IgM, indicating that intrauterine infections are rare [29]. The same study also noted no negative effects on infant health at birth from WNV infection during pregnancy [29]. In some cases, WNV-specific antibodies may appear in cerebrospinal fluid before being detectable in the blood [39]. These findings emphasize the importance of antibody testing in diagnosing WNV and assessing its impact on different groups.

Most newborns were delivered vaginally and at term, while approximately 5–6% were delivered preterm [29,30], in some cases attributable to the onset of pre-eclampsia. Most women who have been infected with WNV during pregnancy have delivered infants without evidence of infection or clinical abnormalities [26,29,30]. However, transplacental transmission of WNV, although rare, can occur in diverse timings throughout pregnancy. WNV tends to infect mouse fetuses more efficiently during the first week of pregnancy than thereafter, implying that different stages of placental development may result in different risks of fetal infection [40]. Similarly, WNV titers in the placenta are detectable and higher earlier after infection than in other maternal organs [40]. The CDC registry tracked pregnancies from 2003 to 2004 and identified 77 women infected with WN virus in 16 states [30]. Of the 72 infants followed, 4% had symptomatic WNV disease at or shortly after birth. These infants were born to women who had developed symptomatic WNV infection within three weeks of delivery. Although cord blood or infant serum at the time of delivery were not available, intrauterine infection or infection at the time of delivery was possible. However, transmission appears to be rare. A retrospective study among 549 infants born after a community-wide epidemic of WNV examined the relationship between birth outcomes and possible exposure to WNV during pregnancy [29]. Cord blood samples looking for immunoglobulin M (IgM) antibodies to the WNV provided no evidence of congenital WNV infections, and there were no significant clinical differences between infants of exposed and unexposed mothers. This comes in sharp contrast with ZIKV infections, where higher rates of transplacental transmission, leading to congenital issues, have been recorded [17].

However, it remains unclear whether occult congenital WNV infection (lacking serologic evidence of infection) could be linked to adverse outcomes [30]. The sensitivity of detecting anti-WNV IgM in cord blood for diagnosing congenital WNV infection is unknown. Limited sensitivity in identifying other congenital infections through specific IgM antibodies suggests that in utero antibody production might be impaired. This could result in congenitally infected infants failing to produce detectable levels of WNV-specific antibodies, particularly if the infection occurred early in pregnancy [30]. In such scenarios, where viral RNA or antigen is also undetectable, the diagnosis of congenital WNV infection would rely on clinical abnormalities. However, the lack of understanding regarding the clinical spectrum of congenital WNV infection complicates this approach.

Regarding neonates, the maternal disease did not generally affect them in terms of clinical manifestations or neurological disorders. Most deliveries proceeded without complications, with neonates typically achieving high Apgar scores. Premature birth and low birth weight were not significantly associated with maternal WNV infection. The majority of neonates exhibited normal weight, height, and head circumference [26,27,28,29,30,31,32]. Some neonates displayed symptoms such as rash and fever immediately after birth, and a few had IgM antibodies against the virus. However, there is no evidence from the included studies that WNV infection during pregnancy results in long-lasting adverse effects on neonates. Most developmental and growth parameters appeared normal [28]. In terms of cognitive development, the majority of children were unaffected by maternal WNV infection, demonstrating normal cognitive development. One child exhibited a moderate delay in adaptability, attributed to environmental factors rather than the virus. Most children were born with normal weight and head circumference, and there was no indication that the virus caused premature births [26,27]. The occurrence of significant deficiencies appeared to correlate with the trimester of gestation during which the mother contracted the virus. Specifically, mothers who contracted the virus in the first trimester had neonates with polydactyly, those infected in the second trimester had neonates with microcephaly and one with Down syndrome, and those infected in the third trimester had neonates with aortic isthmus stenosis, cleft palate, and lissencephaly [30]. Of note, the WNV rarely leads to these severe outcomes, contrary to the ZIKV, where a strong link to congenital abnormalities including microcephaly, brain calcifications, etc. has been observed [17].

Concerning breast milk and colostrum, antibodies or RNA from the virus were detectable. Nevertheless, the data do not conclusively prove transmission of the virus through breast milk. Breastfed infants remained healthy and showed no signs of infection. Antibodies and RNA were typically present in breast milk at the onset of maternal illness and subsequently disappeared [29,30]. Resuming all the included studies regarding congenital transmission, only O’Leary et al. reported three probable congenital infection cases of neonates born to infected symptomatic mothers within three weeks before delivery. All three infants had symptoms of WNV disease, but since there was no serum or cord blood during delivery, it cannot be determined whether they were infected after delivery or transplacentally [30]. Current hypotheses of potential infant transmission in these cases include higher virus inoculum from the blood transfusion or maternal factors present at the time of infection, such as asymptomatic mastitis or unreported minor lesions on the areola, infant age, and respective gastrointestinal maturity, as well as a combination of mother/infant immune factors [28]. This comes in agreement with findings from Zika infection [17]. Both viruses have been detected in breast milk, though transmission through breastfeeding is low for both [41,42]. No significant neonatal infections were reported from breastfeeding with either virus, while infants breastfed by ZIKV-infected mothers may receive maternal antibodies, but the risk of active infection is extremely low [43]. In both cases, mothers are typically encouraged to continue breastfeeding, as the benefits outweigh any minimal theoretical risk [44].

Our review also identified an early study conducted by Sirois et al., where 15 mothers participated along with their children. The findings indicated that the cognitive development of these children was not adversely affected by their mothers’ WNV infection. Among these children, only one was born prematurely, and none had a low birth weight or reduced head circumference. Notably, 82% of the mothers had contracted the WNV during the second or third trimester of pregnancy. Upon completing the Bayley-3 questionnaire, only one child exhibited a moderate delay in adaptability. However, further testing suggested that this delay was attributable to a lack of opportunities in their environment [27]. These conclusions were corroborated by the study conducted by Pridjian et al., which utilized the same assessment tools and found that all children displayed normal or above-average developmental functioning for their age [26].

In our systematic review, significant limitations have been identified. Firstly, there is the issue of publication bias, as all systematic reviews rely on primary studies, and researchers often prefer to publish studies with significant or positive results over those with non-significant findings. Another limitation is the quality of primary studies, which cannot always be controlled. However, we adopted an objective system of assessment of bias. Additionally, there is considerable heterogeneity among the populations studied in each primary research study, the interventions, the outcomes, and the study designs. While this diversity can be beneficial in broadening the scope of our research, it also complicated data comparison and did not allow for solid conclusions to be drawn. Moreover, we only included studies conducted in English in this review, excluding numerous studies published in other languages. Similarly, searching within limited databases may overlook relevant research that could address the research question. Lastly, the limited data available to researchers of the primary studies, as noted in their publications, can further lead to potential errors [26,27,28,29,30,31,32].

It appears that there is a clear necessity for additional research concerning the effects of the West Nile virus on pregnant women and neonates. Such studies are essential to yield comprehensive results, which can subsequently inform the development of guidelines and conclusions. Various study designs, including case reports, pregnancy registries, and cohort studies, have been used to investigate the prenatal effects of WNV infection. However, birth defects surveillance systems have not been employed, as congenital WNV infection lacks a distinct phenotype that would allow for specific identification through such systems [45].

Studies with larger sample sizes are needed in order to finalize the case of WNV during pregnancy and congenital infection due to WNV. Prompt diagnosis, including more robust methods to improve the diagnostic capacity and implementation of routine screening in areas of increased endemicity, is pivotal to identifying cases. Moreover, further preventive measures are crucial in controlling the spread of the WNV. Public health strategies include mosquito control programs, public education on personal protective measures, and surveillance of mosquito populations and avian hosts [45]. The use of insect repellents, protective clothing, and avoiding outdoor activities during peak mosquito activity can reduce the risk of mosquito bites [45]. Climate change and global travel have contributed to the expanding range of WNV, highlighting the need for continued vigilance and adaptive public health strategies. Research into the virology, pathogenesis, and epidemiology of the WNV is essential for the development of effective interventions and mitigation strategies [46]. In summary, the WNV remains a significant vector-borne pathogen with the potential for severe disease outcomes in humans. Data on pregnant populations remain limited. Comprehensive public health measures, combined with ongoing research, are vital for managing the impact of this emerging infectious disease in this special population.

## Figures and Tables

**Table 1:** Newcastle–Ottawa scale.

	**STARS**				
	[25]	[26]	[27]	[28]	[29]
SELECTION					
[A] Truly representative sample for the average of the target population	0	0	0	1	0
[Β] Approximately representative sample for the average target population	1	1	1	1	0
[C] Selected groups of people	1	0	0	0	1
[D] The sampling strategy was not described	0	0	0	0	0
SAMPLE SIZE					
[A] Satisfactory	1	1	0	1	1
[B] Unsatisfactory	0	0	1	0	0
UNRESPONSIVENESS					
[A] Comparison between characteristics of responders and non-responders, and the response rate was satisfactory	1	0	0	1	1
[B] Comparison between characteristics of responders and non-responders was unsatisfactory, and the response rate was unsatisfactory	0	1	1	0	0
[C] No comparison was described between characteristics of responders and non-responders, and the response rate was satisfactory	0	0	0	0	0
ASSESSMENT OF THE RISK FACTOR					
[A] Appropriate measurement tool	1	1	1	1	1
[B] Not an appropriate measurement tool, but was available or described	0	0	0	0	0
[C] Tool not included	0	0	0	0	0
COMPARABILITY [CONTROL OF CONFOUNDING FACTORS]					
[A] Control only on the main factor	1	1	1	1	1
[B] Control on each factor	0	0	0	0	0
OUTCOME					
(1) Evaluation of outcomes					
[A] Independent blind evaluation	0	0	0	0	0
[B] Data matching	0	0	0	0	0
[C] Self-reporting	1	1	1	1	1
[D] Not included	0	0	0	0	0
(2) Statistical test					
[A] Is appropriate	1	1	1	1	1
[B] Not appropriate	0	0	0	0	0

**Table 2:** Summary of the included studies.

Included Studies	Study Design	Parameters Assessed	Clinical Manifestations	Diagnosis/Serology	Outcomes/Conclusions
**Study of frequencies of WNV infections during pregnancy and intrauterine WNV infections**					
Paisley J. E. et al., 2005 (U.S.A.) [29]	Cross-sectional study, 566 women presenting for delivery and 549 newborns	Demographic characteristics, clinical symptoms (fever and timing of fever, WNV infection, congenital infection) and maternal IgG, IgM testing and growth, IgG testing, IgM audiogram, and newborn Apgar scale	Maternal: fever Newborn: 15% Apgar score < 7 5.3% low birth weight, 2.4% short stature, 4.9% smaller head, retinal hemorrhage, Roth spots	22/547 (4%) IgG (+), 0/547 IgM (+) in cord samples in newborns 5/184 Maternal serum IgM (+)/IgG (+) & IgG cord blood (+) but IgM cord blood (−)	- There were no statistically significant differences in neonates according to maternal disease. - Intrauterine WNV infections seemed to be infrequent.
**Study of effects during pregnancy**					
Chapa J. B. et al., 2003 (U.S.A.) [31]	Case study, 1 African-American woman affected by the virus, in the second trimester of pregnancy	Demographic characteristics, clinical symptoms and maternal IgG, IgM control and growth, IgG, IgM control, and newborn Apgar score	Maternal: fever, headache, nausea, vomiting, sore throat, neck stiffness, diplopia, reduced joint movement, preeclampsia	IgM maternal CSF Positive–Serology on newborn not performed	- Pregnant women are at high risk of developing serious complications such as encephalitis. - Data on the effect of WNV on the fetus are limited.
Pridjian G. et al., 2016 (U.S.A.) [26]	Prospective longitudinal cohort study, 28 pregnant serologically confirmed WNV women, matched on maternal age and enrollment month with 25 controls, and their newborns	Pregnancy and newborn data were collected; cord blood WNV serology was obtained. Pediatric exams and the Bayley Scales of Infant and Toddler Development-Third Edition (Bayley-III) were performed	Maternal: fever, rash, low concentration, weakness, meningitis, encephalitis	Maternal IgG 28 (+)/IgM (−)	- No differences in pregnancy and delivery characteristics. - Mothers with infection were more likely to have a fever and used a greater amount of medication. - Birth weight, length, head circumference, and rate of congenital malformations were similar in babies born to WNV-infected and -uninfected mothers. - Follow-up physical exams were generally normal. - The Bayley-III assessments, available for 17 children born to mothers with WNV illness, showed performance at or above age level across domains. - The risk for adverse pregnancy and newborn outcomes in WNV pregnant women appears to be low.
O’Leary D. R. et al., 2015 (U.S.A.) [30]	Cross-sectional study, 71 women with WNV and 72 of their newborns	Demographic characteristics, clinical symptoms and maternal IgG, IgM control and growth, IgG, IgM control, and newborn Apgar score	77 pregnant women with WNV were monitored: 71 delivered 72 live infants, while 4 had miscarriages, and 2 had elective abortions. Most infants were born at term Newborn: 5.6% preterm, 4.8% low birth weight, 10.6% some major birth defect (aortic coarctation, cleft palate, Down syndrome, lissencephaly, microcephaly, and polydactyly)	1/55 (cord serum) infants IgM (+)	- Three infants had a WNV infection that could have been congenitally acquired. - Seven infants had major malformations, but only three of these had defects that could have been caused by maternal WNV infection based on the timing of the infections and the sensitive developmental period for the specific malformations, and none had any conclusive evidence of WNV etiology.
Stewart R. D. et al., 2013 (U.S.A.) [32]	Case study, 3 women with WNV and 1 newborn	Demographic characteristics, clinical symptoms and maternal IgG, IgM control and growth, IgG, IgM control, and newborn Apgar score	Maternal: fever (66.6%), Nausea/vomiting (66.6%), headache (33,3%), neurologic symptoms (33.3%)	3 Maternal IgG (+)/3 IgM (+) − 1/1 newborn IgG (+)/IgM (−)	- The effect of pregnancy on WNV infection and the effect of maternal WNV infection on the fetus both require further investigation.
**Study of effects during lactation**					
Hinckley A. F. et al., 2015 (U.S.A.) [28]	Cross-sectional study, 6 WNV mothers while breastfeeding their infants	IgA, IgM, IgG, WNV Neutralizing Antibody Titer, and SLEV Neutralizing Antibody Titer were studied in breast milk, maternal and child plasma	Newborn: transient papular rash, aortic isthmus stenosis, aortic dissection	Constituents in Milk 1/6 (+) in WNV Neutralizing $Antibody Titer, Constituents in Maternal Serum 5/6 (+) in WNV Neutralizing Antibody Titer, 6/6 IgM(+)/IgG(+) Constituents in Child Serum 0/6 IgM(+)/IgG(+)	- Six infants breastfed from mothers with WNV: five remained healthy, and one developed a rash. - Two fell ill during breastfeeding, and two had congenital antibodies.
**Study of effects on development**					
Sirois P. A. et al., 2014 (U.S.A.) [27]	Cross-sectional study, 11 neonates and infants born to mothers with WNV	Demographic characteristics, growth screening, Bayley scale III, and eye examination of newborns	Νo significant clinical events were reported 1/11 newborns were born prematurely. While completing the questionnaire, 1/11 children had a moderate developmental delay	Infant and neonate IgG n/IgM not measured	- Maternal WNV infection does not appear to be associated with global developmental delays in young children.

## Data Availability

Data are contained within the article.
